# Thanks to Editorial Board of Arquivos de Neuro-Psiquiatria

**DOI:** 10.1055/s-0044-1782631

**Published:** 2024-03-14

The Editors-in-Chief and Deputy Editors of Arquivos de Neuro-Psiquiatria extend their gratitude to the Associate Editors ( **68** ), Editorial Board ( **50** ), and Referees ( **317** ) who altruistically dedicated their time to the editorial tasks of this journal from **January 1st, 2023** , to **December 31st, 2023** . We appreciate your invaluable contributions.

Thank you very much!

1. Abimael González-Hernández
2. Acary Souza Bulle Oliveira
3. Adalberto Studart-Neto
4. Adriana Carra
5. Adriana Moro
6. Alan Flores
7. Alberto David Fadul Filho
8. Alberto J. Espay
9. Alberto Rolim Muro Martinez
10. Alessandro Finkelsztejn
11. Alex Meira
12. Alexandra Prufer de Queiroz Campos Araújo
13. Alexandre Maia Barros
14. Aline Mourao
15. Amilton Santos
16. Amnuay Kleebayoon
17. Ana Carolina Coan
18. Ana Claudia Souza
19. Ana Paula Etges
20. Ana Vigil Martínez
21. Anderson Paiva
22. Andre Felicio
23. André Palmini
24. André Silva
25. Andrea Cappellano
26. Andresa Braga Baiak
27. Angel Vistorte
28. Arthur Poetscher
29. Artur Coutinho
30. Artur Schumacher-Schuh
31. Aylin Bican Demir
32. Bárbara Panichelli
33. Beatriz Casella
34. Beatriz Mendes
35. Bernd Holdorff
36. Bruno Pedreira
37. Bruno Rodrigues
38. Caio Disserol
39. Carlo Domênico Marrone
40. Carlos Alberto Guerreiro
41. Carlos Bernardo Tauil
42. Carlos Otto Heise
43. Carlos Roberto Rieder
44. Carolina de Medeiros Rimkus
45. Carolina Rouanet
46. Cassio Meira
47. Ceres Ferretti
48. Clara Barreira
49. Clarice Listik
50. Conrado Estol
51. Cristiana Borges Pereira
52. Cristiane Moreno
53. Cristiane Nascimento Soares
54. Cybelle Diniz
55. Dalva Lucia Rollemberg Poyares
56. Daniel San-Martin
57. Danielle Bruno
58. Davi Solla
59. Debora Maia
60. Débora Palma Maia
61. Delson José Silva
62. Denise Pinheiro
63. Denise Sisterolli Diniz
64. Diogo Santos
65. Eberval Gadelha Figueiredo
66. Eduardo Estephan
67. Eduardo Mutarelli
68. Eduardo Nogueira
69. Eduardo Ribas
70. Eduardo Yamaguchi
71. Elcio Juliato Piovesan
72. Eliana da Cunha
73. Eliana Melhado
74. Elisa de Melo
75. Elisa Resende
76. Eliseu Paglioli
77. Elmano Carvalho
78. Emilia M. Gatto
79. Erasmo Barbante Casella
80. Erdal İn
81. Eric Hargreaves
82. Erika Treptow
83. Erildo Vicente Muller
84. Eva Rocha
85. Fabiano Moulin
86. Fabio Ejzenbaum
87. Fabio M. Iwamoto
88. Fabíola Dach
89. Farmy Silva
90. Felipe Pacheco
91. Felipe Saba
92. Felipe von Glehn
93. Fernanda Carvalho
94. Fernando Cendes
95. Fernando Dantas
96. Fernando Morgadinho Coelho
97. Fernando Stelzer
98. Flávia Rolim
99. Flávio Rezende Filho
100. Francisco de Assis Aquino Gondim
101. Francisco Dias
102. Francisco Luciano Pontes Junior
103. Franz Onishi
104. Frederico Moura
105. Gabriel Braga
106. Gabriel Cismaru
107. Gabriela Cipolli
108. Gabriela Moreira
109. Geraldo Rizzo
110. Giordani dos Passos
111. Gisele Sampaio Silva
112. Gloria Maria Tedrus
113. Guilherme Diogo Silva
114. Gustavo Franklin
115. Gustavo Luvizutto
116. Gutemberg dos Santos
117. Henrique Ferraz
118. Henrique Guimarães
119. Henrique Salmazo da Silva
120. Hinpetch Daungsupawong
121. Hsin Chien
122. Igor Pagiola
123. Isabela Bensenor
124. Isabella D'Andrea Meira
125. Jaime Lin
126. Jaison Antonio Barreto
127. Jay Mohr
128. Jefferson Becker
129. João Aris Kouyoumdjian
130. João Brainer Clares Andrade
131. João Eudes Magalhães
132. João Renato Rebello Pinho
133. Jonas Jardim de Paula
134. Jorge Bevilacqua
135. José Garbino
136. Jose M. Ferro
137. José Marcelino Aragão Fernandes
138. José Roberto Tude Melo
139. Jose Testa
140. Josemir Sander
141. Juan Rojas
142. Juliana Gurgel-Gianetti
143. Juliana Valentim Bittencourt
144. Jussara Baggio
145. Jussimara Monteiro
146. Karen Nunez-Wallace
147. Katia Lin
148. Kazuo Nakamichi
149. Kelson Almeida
150. Kenia Campanholo
151. Kristofer Ramina
152. Larissa Salvador
153. Laura Lopes
154. Lea Tenenholz Grinberg
155. Leandro Tavares Lucato
156. Leonardo Carbonera
157. Leonardo Cruz de Souza
158. Leonardo Welling
159. Letícia Pereira de Brito Sampaio
160. Lidianne Neri
161. Lineu César Werneck
162. Lorena Viana
163. Lucas Mourão
164. Lucia Iracema Zanotto Mendonça
165. Luciana Barbosa
166. Luciana de Resende
167. Luciana León Cejas
168. Luciana Neves
169. Luciano Drager
170. Luís dos Ramos Machado
171. Luís Felipe Ravic de Miranda
172. Luís Otávio Sales Ferreira Caboclo
173. Luiz Eduardo Betting
174. Luiz Paulo Queiroz
175. Magda Lahorgue Nunes
176. Maira Barbosa
177. Maira Olchik
178. Mamede de Carvalho
179. Marcel Leal Ribeiro
180. Marcela Cordellini
181. Marcela Freitas
182. Marcela Mansur-Alves
183. Marcelo Aragão
184. Marcelo Caetano
185. Marcelo Masruha Rodrigues
186. Marcelo Schafranski
187. Márcia Ribeiro
188. Marcio Nattan Souza
189. Marco Antonio Araujo Leite
190. Marco Antonio Lima
191. Marco Aurelio Fornazieri
192. Marco Aurélio Lana-Peixoto
193. Marcondes Cavalcante França Junior
194. Marcos Christiano Lange
195. Marcos Gomes de Freitas
196. Marcos R. G. Freitas
197. Marcus Della Coletta
198. Marcus Vinícius Pinto
199. Maria Carolina Pedro Athie
200. Maria Joana Mader Joaquim
201. Maria Lucia Vellutini Pimentel
202. Mariana Artilheiro
203. Mariana Spitz
204. Marianna de Moraes
205. Mário Fernando Prieto Peres
206. Mário Luciano de Mélo Silva Júnior
207. Mario Sato
208. Marlon Aliberti
209. Marlon Wycliff Caeira
210. Martin J. Brodie
211. Martin Underwood
212. Mateus Boaventura
213. Mateus Nogueira
214. Matt Parton
215. Maurizio Elia
216. Michaei Firmage
217. Michael Hornberger
218. Milena Sales Pitombeira
219. Millene Camilo
220. Monica Levy Andersen
221. Myriam Bonadiu Pelosi
222. Nadia Shigaeff
223. Natalia Trombini Mendes
224. Nathália Bergamasco
225. Nathália de Figueiredo
226. Nathane Braga
227. Neide Alonso
228. Neslihan Eskut
229. Nicollas Nunes Rabelo
230. Nilton Custodio
231. Norberto Frota
232. Orlando Graziani Povoas Barsottini
233. Otto Fustes
234. Paul Delano
235. Paulo Caramelli
236. Paulo Christo
237. Paulo Henrique Bertolucci
238. Paulo José Lorenzoni
239. Paulo Victor de Souza
240. Pedro André Kowacs
241. Pedro Braga-Neto
242. Pedro Brandão
243. Pedro Genta
244. Pedro Kowacs
245. Pedro Tomaselli
246. Priscilla Avelino Ferreira Pinto
247. Rachael Rodrigues
248. Radhika Nair
249. Rafael Vieira
250. Rafael Carneiro
251. Ramesh Kandimalla
252. Renan Castello Branco
253. Renan Domingues
254. Renata Kochhann
255. Renata Londero
256. Renata Siciliani Scalco
257. Renato Arruda
258. Renato Puppi Munhoz
259. Ricardo Brito
260. Ricardo Nitrini
261. Ricardo Sukiennik
262. Rita Raisman-Vozari
263. Roberta Arb Saba
264. Rodrigo Bazan
265. Rodrigo Brisson
266. Rodrigo Kleinpaul
267. Rogério Aires
268. Rogerio de Oliveira
269. Ronaldo Abraham
270. Rubens Cury
271. Rubens José Gagliardi
272. Saitin Sim
273. Sâmia Wayhs
274. Samir Magalhães
275. Sarah Camargos
276. Sebahattin Karabulut
277. Sebastián Ameriso
278. Sérgio Brasil
279. Sheila Trentin
280. Slaven Pikija
281. Suzete Farias da Guarda
282. Sylvia Ciasca
283. Tamine Capato
284. Tânia Bovolenta
285. Tarso Adoni
286. Tatiana de Oliveira
287. Tatiana Luft
288. Teresa Torralva
289. Thais Bento Lima da Silva
290. Thiago Macedo e Cordeiro
291. Thiago Vale
292. Valeria Scavasine
293. Vanderson Neri
294. Vanessa Holanda
295. Vanessa Toscano
296. Veronica Aran
297. Victor Hugo Marussi
298. Victor Rivera
299. Vineet Punia
300. Vinícius Braga
301. Vinicius Cota
302. Vinicius Montanaro
303. Vinicius Schoeps
304. Vítor Tumas
305. Viviane Carvalho Crelier
306. William B. Rizzo
307. William Whitehouse
308. Wilson Marques Jr
309. Wladimir Bocca Vieira de Rezende Pinto
310. Ylmar Correa-Neto
311. Yu Jiang
312. Yuri M. Costa
313. Zahide Mail Gürkan
314. Zeferino Demartini Jr
315. Zeynep Atay

